# A pilot study to assess the feasibility and impact of a brief motivational intervention on problem drug and alcohol use in adult mental health inpatient units: study protocol for a randomized controlled trial

**DOI:** 10.1186/1745-6215-15-308

**Published:** 2014-08-01

**Authors:** Hermine L Graham, Max Birchwood, Emma Griffith, Nick Freemantle, Paul McCrone, Chrysi A Stefanidou, Kathryn Walsh, Latoya Clarke, Arsal Rana, Alex Copello

**Affiliations:** University of Birmingham, Edgbaston, Birmingham B15 2TT UK; Division of Mental Health and Wellbeing, Warwick Medical School, University of Warwick, CV4 7AL Coventry, UK; University of Bath, Claverton Down Bath, BA2 7AY UK; Department of Primary Care and Population Health, Upper Third Floor, UCL Medical School (Royal Free Campus), Rowland Hill Street, London, NW3 2PF UK; Health Service and Population Research Department, Institute of Psychiatry, Kings College London, London, UK; University of Birmingham, Edgbaston, B15 2TT Birmingham UK

**Keywords:** Inpatient, Comorbid, intervention, Brief motivational intervention, Treatment, Randomized controlled trial, Dual diagnosis, Substance misuse, Severe mental health condition, Schizophrenia, Psychiatric hospital admission

## Abstract

**Background:**

Substance misuse in those with severe mental health problems is common and associated with poor engagement in treatment and treatment outcomes. Up to 44% of those admitted into psychiatric inpatient facilities have coexisting substance-misuse problems. However, this is not routinely addressed as part of their treatment plan. A mental health admission may present a window of opportunity for inpatients to reevaluate the impact of their substance use. This study will aim to evaluate the effectiveness of a targeted brief motivational intervention in improving engagement in treatment and to assess how feasible and acceptable this intervention is to inpatients and staff as a routine intervention.

**Methods/Design:**

This randomized controlled trial will use concealed randomization; blind, independent assessment of outcome at 3 months; characterization of refusers and dropouts; and be analyzed according to the intention-to-treat principle. After baseline assessments, eligible participants will be randomized either to the Brief Integrated Motivational Intervention plus Treatment As Usual, or Treatment as Usual alone. Eligible participants will be those who are new admissions; >18 years; ICD-10 diagnosis of -schizophrenia or related disorder, bipolar affective disorder, recurrent depressive disorder, and DSM-IV diagnosis of substance abuse or dependence over the last 3 months. The primary outcome is engagement in treatment for substance misuse, and secondary outcomes include readiness to change substance misuse together with a cost-effectiveness analysis. Qualitative interviews with staff and participants will assess the acceptability of the intervention.

**Discussion:**

This pilot randomized trial will provide the first robust evidence base for inpatient care of people with severe mental health problems and co-morbid substance misuse and provide the groundwork for confirmatory trials to evaluate a potentially feasible, cost-effective, and easy-to-implement treatment option that may be readily integrated into standard inpatient and community-based care.

**Trial registration:**

ISRCTN43548483 Date of ISRCTN assignation: 4/17/2014.

## Background

### Substance misuse in mental health-admission units

It is now well recognized that substance misuse in those with severe mental health problems is common [1–4], associated with poor engagement in treatment and treatment outcomes [5–7] and low motivation to change drug and alcohol use [8–10]. However, poor treatment engagement is a barrier for change and good treatment outcomes [6, 10–12]. In addition, substance misuse among this population is also associated with increased psychiatric hospital admissions and is found to affect inpatient stays negatively [13]. In the UK, 22% to 44% of those admitted into psychiatric inpatient facilities for mental health problems have been found also to have coexisting alcohol or drug problems [13]. Policy guidance has pointed to the need to train staff to improve routine assessment and treatment of substance misuse as part of the clinical-management strategy of a psychiatric admission [13]. Nonetheless, this has continued to be highlighted as a significant gap in service provision, and outcomes for this group remain poor [14, 15].

As acute symptoms of mental ill health decline, this can be characterized as a time of contemplation and a window of increased awareness and insight into the factors that contributed to becoming mentally unwell and or being admitted into hospital [16, 17]. However, such increased insight may result in increased emotional distress, and research has shown that after discharge, some individuals may “seal over” the experience, in an attempt to reduce emotional distress [18]. That is, they may deny or minimize recent mental health symptoms or experiences and precipitating factors, and as a result, lose awareness of the triggers for becoming unwell [18].

Sealing over the experience of relapse was found to predict low engagement with mental health services 6 months after discharge for psychiatric inpatients [18]. However, engagement in treatment is found to be a key in improving treatment outcomes [10, 19]. Hence, we propose that a targeted brief motivational intervention could use this “window of opportunity” to increase the focus on the role drug and alcohol use may have played in the exacerbation of mental health symptoms and actively link participants with substance-misuse treatment routinely integrated within community mental health teams. Hence a mental health admission may well represent a window of opportunity for individuals who misuse substances to be offered treatment to help them reevaluate their drug/alcohol use and become aware of the negative impact of it on their mental health [13, 14, 16].

Inpatient policy guidance recommends that staff be trained in simple approaches to improve motivation and encourage change in substance use [13]. A recent Cochrane review [20] indicated mixed findings in trials of the effectiveness of long-term psychological interventions with psychosis and substance misuse, potentially because of variations in methodology and samples. However, it points to the need to evaluate brief interventions, such as motivational interviewing, to enable service providers to identify a cost-effective and easy-to-implement component that may be quickly integrated into standard care. Brief motivational interventions have demonstrated good results across the range of substances, often equivalent to longer-term interventions, across a range of populations [21–25]. Motivational interventions seek to change views about alcohol/drug use or drug/drinking behavior. This would be seen as an initial step in promoting long-term changes in behavior.

The evidence base regarding the use of motivational-based interventions with this client group is encouraging [10, 26–29]. However, the evidence suggests that brief interventions be provided within the context of a comprehensive package of ongoing integrated treatment. Thus the aim of this trial is to target drug and alcohol misuse among inpatients with severe mental health problems to determine whether offering a Brief Integrated Motivational Intervention (MI) to inpatients will lead to greater engagement in substance-misuse treatment and motivation to change substance misuse in community services after discharge.

### Hypotheses

The primary hypothesis to be evaluated is whether engagement in treatment for substance misuse can be significantly improved by the MI provided in the context of treatment as usual (TAU). The secondary hypotheses are that those receiving the MI will show greater readiness to change substance-use behavior than those receiving treatment as usual and that the MI + TAU will be more cost-effective than the TAU.

## Method/Design

The trial is funded by the National Institute for Health Research-Research for Patient Benefit and has received ethical approval from the West Midlands–The Black Country National Research Ethics Committee (REC reference: 12/WM/0369). It is a single (rater) blind, intention-to-treat analysis, prospective randomized trial to assess the feasibility and impact of a brief motivational intervention for drug and alcohol misuse in mental health admission units. The trial uses concealed randomization; blind, independent assessment of outcome at 3 months; characterization of refusers and dropouts; and analysis by intention to treat. Participants consented and then randomized on a 1:1 basis, to one of two experimental conditions: MI in the context of Treatment As Usual or TAU. Participants being recruited are adults aged 18 years or older with schizophrenia, schizoaffective or delusional disorders, bipolar affective disorders or recurrent depressive disorder, users of community mental health services; new admissions to six mental health inpatient units in the West Midlands within the acute phase of severe mental health problems; who are also misusing substances (alcohol and/or drugs). Participants are recruited from six inpatient units, including 11 acute wards and three Psychiatric Intensive Care Units (PICUs), which offer a total of 202 beds.

Recruitment to the trial began in April 2013 and is ongoing until July 2014. Follow-up assessments began in May 2013 and will be completed by September 2014.

### The intervention

The MI is offered in the context of TAU. The MI seeks to encourage participants to engage in talking about their substance use and its impact on their mental health, the fundamental first step in the process of promoting a readiness and willingness to change problematic drug/alcohol use. The initial aim is twofold; first, to increase awareness of the advantages and the disadvantages of continued substance misuse, and second, to build awareness of the impact of substance use on mental health.

The next stage encourages participants to contemplate change and make a change plan, thereby making change feel possible. Participants are provided with individually tailored psychoeducational material about substances and, between sessions, are also encouraged to access websites offering information about alcohol (”Down your Drink”) and drugs (”Talk to Frank”). Participants are offered a Peer Mentor after the first week of the intervention. The Peer Mentor will aim to show empathy and understanding during a difficult time for the participants, to share personal experiences, to offer an alternative outlook on problematic substance use, and to provide some support and solidarity.

The MI is based on specifically trained staff on the wards working alongside staff from the specialist COMPASS Programme team (a specialist “dual diagnosis” Trust-wide service) [30]. Their aim is building good collaborative relationships with participants working toward a joint goal of “keeping participants from returning to hospital”. The MI takes place over a 2-week period for four to six sessions for 15 to 30 minutes on each occasion. At the final session of the 2-week intervention, the booster session is arranged for a month.

The booster session is provided by a member of the specialist COMPASS Programme Team and attended by the participant’s Care Co-ordinator. It will seek to help consolidate motivation and transfer the skills from the MI to the community and to link participants actively with substance-misuse treatment routinely integrated within community mental health teams.

The structure of the MI attempts to map itself onto the stage of recovery in acute psychosis in a targeted manner. It targets the initial window of contemplation during the admission and then is timed to coincide with just before “sealing over” and disengagement [16, 18] are predicted to occur. The MI is delivered jointly by a staff member on the unit and a member of the specialist COMPASS Programme team [30], according to a therapy manual designed for this purpose. Staff are trained and supervised in the delivery of the MI by three of the Investigators. The standard to which staff deliver the MI is regularly monitored and assessed for fidelity and adherence.

### Treatment as usual

Treatment as usual will be documented. It is mostly provided by nursing and medical staff on the mental health admission units in line with inpatient trust policies, regularly monitored by the UK Care Quality Commission. It will primarily consist of assessment and monitoring mental state, provision of medication, and stabilization of the mental state.

### Inclusion and exclusion criteria

Eligible participants are adults aged 18 or older with severe mental health problems, who are service users of community mental health services and have been admitted to mental health inpatient units within the acute phase of severe mental health problems and who are also misusing substances (alcohol and/or drugs). They will have an ICD-10 diagnosis of schizophrenia, schizoaffective or delusional disorders (F20,22,23,25,28,29); bipolar affective disorders (F31); recurrent depressive disorder without psychotic symptoms (F33.2) (32), and be identified as misusing alcohol and/or drugs over the past month, in addition to a minimum score of 3 (abuse/dependent use based on DSM-IV diagnostic criteria for substance-related disorders) on the Clinicians Alcohol/Drugs Use rating scale over the past 3 months [31]. They also must be assessed by the Responsible Clinical Officer as having capacity to consent, and have a Care Co-ordinator in a Community Mental Heath Team. Participants who have already been entered in the trial but are re-admitted to the inpatient unit are not screened again but continue to be treated as trial participants and monitored as per protocol.

### Recruitment and randomization

Eligible participants are identified by Research Associates in conjunction with Clinical Studies Officers (CSOs) from the Mental Health Research Network, who review care records. The Research Associates complete a screening measure with Care Co-ordinators to confirm eligibility for the trial. The Research Associates then visit eligible participants within 2 weeks of admission, once the acute symptoms have been assessed as eased and the inpatient is deemed able to provide informed consent. Eligible participants are invited to take part and asked to provide informed consent.

Once informed consent to participate in the study has been obtained, the researchers administer a battery of assessments, and on completion, participants are randomly allocated either to the MI group (in the context of TAU) or the TAU group (the control group). At the end of the baseline assessment session, the researchers schedule a meeting with the participant for 2 weeks’ time, to complete the posttreatment assessments. Just before the 3-month data-collection point, the researchers contact the Care Co-ordinator to schedule a meeting for the completion of the 3-month follow-up assessment battery.

The trial uses independent central randomization by using a concealed process via Email. Both researchers are blind to participant treatment group allocation until all baseline, posttreatment, and 3-month follow-up quantitative assessments have been completed. Treatment allocation is available to the researchers once the 3-month data-collection point (primary outcome) has been completed. Participants in the MI group then complete the qualitative interview.

Data also are collected regarding the total number of participants admitted to the inpatient unit, the number who use substances, and the number who are eligible and consent to participate in the study.

Given the pragmatic design of the trial and the nature of inpatient units, it is possible that some leakage of MI components into TAU may occur through participants or staff. Participants may inadvertently discuss or share information about the MI, or inpatient staff trained to use the intervention may provide parts of it to those participants in the TAU arm. Any contamination bias will be limited by steps taken by the trial team. A number of methods will be used to reduce potential leakage. Participants will be informed of the nature of the study during the consenting process and asked not to share any information about the treatment they receive. An important component of the training sessions for Inpatient staff and Peer Mentors in the intervention arm will involve education and awareness about the importance of blinding and ensuring that only those allocated to the MI receive the elements of the intervention. This is also to be discussed during supervision sessions.

### Measures

#### Primary outcome

Primary outcome will be engagement with substance-misuse treatment while inpatients and with community treatment services at 3-month follow-up, as reported by Care Co-ordinators/primary clinician, by using the Substance Abuse Treatment scale [8, 31]. Engagement is assessed by using the Substance Abuse Treatment Scale (SATS), a widely used and standardized measure that uses an eight-stage hierarchic motivational model of engagement in substance-misuse treatment to assess the stage of recovery from substance misuse.

Care Coordinators are asked to categorize the level of engagement in substance-misuse treatment on an 8-point scale: the categories are pre-engagement, engagement, early persuasion, late persuasion, early active treatment, late active treatment, relapse prevention, and in remission or recovery. It has demonstrated high validity, reliability, and test-retest reliability [8, 31]. The reporting timeframe is adapted to the previous 3 months. An objective assessment of engagement in addressing substance misuse will also be carried out by reviewing the clinical notes of participants to assess whether any discussion of substance use has occurred, as evidenced in the clinical notes of participants from both the MI and TAU groups.

#### Secondary outcomes

##### i) Motivation to change

Readiness to change alcohol and drug use is assessed by using the Stages Of Change Readiness And Treatment Eagerness Scale (SOCRATES). The measure consists of 19items that assess current readiness for change. The three factors derived from the scale are Recognition of substance use as being problematic, Ambivalence, and Taking Steps toward change. It has been widely used and has demonstrated good validity and reliability [32].

The Importance-Confidence Ruler is a global assessment of level of motivation and confidence to change that assesses two concepts that are suggested to underpin readiness to change [33]. Participants are asked to rate on a scale of 0 to 10 how important it is to change the use of the specified substance and how confident they are that they will succeed.

##### ii) Drug Use and Alcohol Use

Drug and alcohol use will be assessed by using a number of complementary measures.

The Clinicians Alcohol/Drugs Use rating scales (CDUS/CAUS) have been developed, based on DSM-IV diagnostic criteria for substance-related disorders, and are used by primary clinicians to classify reliably the severity of substance use among people with severe mental health problems [31]. The measure has demonstrated sensitivity and high levels of reliability [31]. The reporting time frame is the previous 3 months.

Quantity and pattern of drug and alcohol use is assessed with participants by using the drug-use profile section B of the Maudsley Addiction Profile (MAP) [34]. This addresses the number of days each substance has been used in the past 30 days, the average amount of use of each drug on a using day, and the number of days of injecting drug use. The MAP is a widely used tool that has demonstrated good validity and high test-retest reliability [34]. Information on the age at onset of use of each substance also is collected.

The Severity of Dependence Scale is a validated and widely used measure for screening the severity of dependence, based on self-report [35]. It was constructed for use with various substances. The scale consists of five items, each rated on a 4-point Likert scale. A score over 4 constitutes dependency. The items focus on the individual’s anxiety, preoccupation, and control over use of the drug. It has high reliability across a range of samples [35–37].

The Alcohol Use Disorders Identification Test (AUDIT) is a well-established measure, developed by the World Health Organisation [38] to assess patterns of use and the impacts of use. It is a 10-item self-report questionnaire investigating alcohol consumption, scored from 0 to 4 for each question, giving a maximum score of 40. Scores of 8 or more are associated with harmful or hazardous drinking, and a score of 13 or more in women, and 15 or more in men, is likely to indicate alcohol dependence. The AUDIT questionnaire has been found to have high levels of reliability [39].

##### iii) Psychological Functioning

Psychological functioning is measured by using the Recovery Style Questionnaire (RSQ), Insight Scale, and the Hospital Anxiety and Depression Scale (HADS). The RSQ is a 39-item measure designed to assess two concepts of recovery; Integration and Sealing-over. It is a self-report measure that consists of 13 subscales, which allows six recovery styles to be classified based on a continuum (sealing over; tending toward sealing over; mixed-picture predominantly sealing over; mixed picture predominantly integration; tending toward integration; integration. The RSQ has demonstrated excellent psychometric properties [40].

The Insight Scale (IS) is an eight-item self-report scale that sensitively assess changes in levels of insight in terms of perceived need for treatment, relabeling symptoms as problematic, and awareness of illness. It is a widely used scale that has demonstrated good validity and reliability [41].

The HADS is a well-established 14-item self-report measure that has been found to assess anxiety and depression reliably. It has demonstrated excellent psychometric properties [42, 43].

The process variable that will be measured will be perceived importance and confidence to change substance use by using the Importance-confidence ruler [33].

### Cost-effectiveness analysis

Principal data on costs and resource use will be collected prospectively alongside the trial. The intervention costs will be calculated by using information on staff grade, training and supervision required, and overheads. The use of services during the inpatient stay and in the community up to follow-up will be measured by using the Client Service Receipt Inventory [44]. These data will be combined with appropriate unit costs (NHS Reference Costs, PSSRU data) to calculate care costs. The EQ-5D [45] is commonly used in economic evaluations to generate QALYs. Costs will be compared between the two groups by using bootstrapping methods to take account of expected skewness. Costs will be combined with QALYs, and incremental cost-effectiveness ratios constructed. Uncertainty around these will be assessed by using cost-effectiveness planes, and further interpretation will be aided by constructing cost-effectiveness acceptability curves.

### Acceptability and feasibility

Qualitative interviews after the 3-month follow-up point, by using a semistructured interview, will seek to establish satisfaction with the treatment received and perceived processes of change, including helpful aspects of the therapeutic process. We will aim to understand which elements of the MI were beneficial and acceptable in the care of people with combined mental health and substance-misuse problems. We will also interview a sample of therapists in focus groups to establish acceptability of the MI and how it differed from standard treatment. This will complement the analysis of the quantitative data and identify ways in which the MI may need to be adjusted in preparation for a definitive trial. Each interview will be recorded and transcribed.

### Analysis

#### Sample size

A power calculation was carried out based on a previous study [46] by using the primary outcome measure SATS. Allocating 68 participants by a 1:1 strategy between the treatment and control conditions (34 participants per group) will have 90% power (1-β) to find a difference of 1 point on the SATS scale to be statistically significant, by using a conventional two-sided α of 0.05. A 1-point difference would be clinically important for participants and mental health services, as it would indicate increasing levels of engagement in treatment and addressing substance misuse (for example, a shift from “Pre-engagement” to “Engagement”).

#### Planned analyses

We describe the characteristics of included subjects categorized by randomized group. The principal analysis will be conducted by using the intention-to-treat principle, based on all randomized patients. The effect of treatment will be estimated by using generalized linear modeling with an identity link and gaussian error. The principal analysis uses the SATS score at follow-up as the response variable and will include the randomized group and the baseline SATS value as a patient-level covariate. Results will be described as a difference in mean SATS value between randomized groups with 95% confidence intervals and corresponding *P* values. All available practical steps will be taken to avoid missing data; however, in this patient population, it is possible that we will be unable to include data from a small number of subjects. We will explore the potential implications of missing data by using regression-based multiple imputation techniques.

Analysis of secondary variables will be conducted by using an analogous approach to that applied to the primary outcome. We will conduct additional exploratory analyses to examine the extent to which the primary outcome is affected by the length of history of involvement with services, and other relevant patient-level characteristics. We will explore potential therapist effects in the intervention group by using generalized random intercept models.

Costs will be compared between the two groups by using bootstrapping methods to take into account the expected skewness. QALY gains will be measured with the EQ-5D. Spearman correlations will be produced to show the strength of the relation between QALY gains and also treatment engagement and willingness to change measures.

For the qualitative analyses, each interview will be recorded and transcribed, where consent is given to record; otherwise, detailed notes will be taken. Transcripts produced will be analyzed by using grounded theory-based methods to identify themes that will be combined to develop a model to understand participants’ and therapists’ perceptions of the treatment.

## Discussion

This pilot randomized trial will be the first testing of an intervention during inpatient care of people with severe mental health problems and comorbid substance misuse. If successful, the trial will provide the groundwork for confirmatory trials to evaluate a potentially feasible, cost-effective, and easy-to-implement treatment option that may be readily integrated into standard inpatient and community-based care.

## Trial status

Ongoing.

## Abbreviations

ICD-10: International Classification of Disorders, 10th Edition
DSM-IV: Diagnostic and Statistical Manual of Mental Disorders, Fourth Edition
UK: United Kingdom
MI: Brief Integrated Motivational Intervention
TAU: treatment as usual
PICU: Psychiatric Intensive Care Unit
COMPASS Programme team: Combined Psychosis and Substance Misuse, a specialist “dual diagnosis” Trust-wide service
CSO: Clinical Studies Officer
SATS: Substance Abuse Treatment Scale
SOCRATES: Stages Of Change Readiness And Treatment Eagerness Scale
CDUS/CAUS: The Clinicians Alcohol/Drugs Use rating scales
MAP: Maudsley Addiction Profile
AUDIT: The Alcohol Use Disorders Identification Test
RSQ: Recovery Style Questionnaire
HADS: Hospital Anxiety and Depression Scale
IS: The Insight Scale
EQ-5D: EuroQol – 5 Dimensions
QALYs: Quality-adjusted life-year.
